# A Comparative Study of Ultrasound-Guided Continuous Adductor Canal Block With Ultrasound-Guided Continuous Femoral Nerve Block in Unilateral Total Knee Arthroplasty for Limb Mobilization and Analgesic Efficacy

**DOI:** 10.7759/cureus.22904

**Published:** 2022-03-06

**Authors:** Raziullah Siddiqui, Sumit Bansal, Arun Puri, Manoj Sinha

**Affiliations:** 1 Anaesthesia, Dr. Hedgewar Aarogya Sansthan Hospital, New Delhi, IND; 2 Anesthesia, Max Super Speciality Hospital, Patparganj, New Delhi, IND

**Keywords:** analgesic efficacy, patient-controlled analgesia, limb mobilization, unilateral total knee arthroplasty, femoral nerve block, adductor canal block

## Abstract

Background

Total knee joint replacement surgery is associated with severe postoperative pain and is amenable to regional anesthesia techniques for pain control. Femoral nerve block (FNB) provides effective analgesia after total knee arthroplasty (TKA) but has been associated with delayed ambulation due to quadriceps muscle weakness. Adductor canal block (ACB) may be a promising alternative, with less effect on the quadriceps muscle and comparable analgesic efficacy. We evaluated the effectiveness, safety, and patient satisfaction of continuous ACB augmented with infiltration between the popliteal artery and capsule of the knee (iPACK) block and compared them with those of continuous FNB amplified with iPACK block in preventing postoperative pain among patients undergoing unilateral total knee replacement (TKR) surgeries.

Methodology

According to a computer-generated sequence from September 2019 to June 2020, 50 American Society of Anesthesiologists grades I-III patients aged between 35 and 75 years who underwent unilateral TKR surgery were randomized into two equal groups, namely, ACB and FNB. The Timed Up and Go (TUG) and 10-minute walk tests were used to detect early ambulation (impact on quadriceps muscle). The secondary goal was to evaluate and compare opioid consumption and analgesic efficacy between the groups measured using a numeric rating scale (NRS). The demographic characteristics, technical difficulty, efficiency, safety, and comfort were compared between the two groups.

Results

During the postoperative period, patients in the ACB group could perform all TUG tests significantly faster than those in the FNB group. The mean get-up time in the ACB group was 39.08 ± 5.53 seconds, whereas that in the FNB group was 44.92 ± 7.10 seconds (p < 0.01). The 3-m walk time was 123.16 ± 15.90 seconds in the ACB group and 134.68 ± 13.13 seconds in the FNB group (p < 0.01). The 10-m walk time was 221.24 ± 18.82 seconds in the ACB group and 245.24 ± 21.68 seconds in the FNB group (p < 0.001). No significant difference was observed in NRS scores between the groups after the first 24 hours. The number of opioids available for consumption in both groups was equivalent.

Conclusions

ACB when augmented with an iPACK block is a good alternative to FNB for unilateral TKR surgeries. ACB may promote early ambulation without a reduction in analgesia when compared with FNB.

## Introduction

Total knee replacement (TKR) is a common orthopedic procedure and is the gold standard treatment for patients with end-stage knee osteoarthritis who have not responded to other treatments. The number of joint replacement surgeries is rising to improve the quality of life owing to increasing life expectancies, sedentary lifestyles, obesity, and advances in healthcare insurance infrastructure and technology. According to a Frost and Sullivan survey (2011), approximately 70,000 hip and knee implants are performed in India each year, with a 25% increase expected over the next five years [1]. Nerve block, epidural catheterization, ultrasound-guided femoral nerve block (FNB) injection and catheterization, and adductor canal block (ACB) and catheterization are among the common practices. Epidural catheterization has bilateral limb effects with delayed bladder movement and has various systemic side effects. FNB is consistently linked to decreased quadriceps muscle strength, with a 2% increase in the probability of falling. Therefore, FNB’s goal of pain alleviation comes at the expense of the maintenance of muscle strength. The ideal nerve block for total knee arthroplasty (TKA) should provide adequate analgesia while preserving muscle power to expedite recovery.

Each analgesic approach has its set of benefits and drawbacks. Patient-controlled analgesia (PCA) using opioids, epidural analgesia (EA), ACB, and FNB with numbing cream infiltration or infusion are all systematic methods in addition to non-steroidal anti-inflammatory drugs. The side effects of PCA opioids are respiratory depression, nausea, urine retention, and constipation. When combined with an anticoagulant, EA is linked to spinal epidural hematoma, hypotension, urine retention, and pruritus [2]. EA can result in bilateral motor blockage, making early mobilization difficult [3]. Moreover, there is an indication that epidural blocking increases the risk of major neurological problems (nerve injury) in patients undergoing TKA [4]. FNB is more effective in controlling pain and has fewer opioid-related side effects than PCA and EA [5,6]. Moreover, anesthesiologists’ interest in peripheral nerve block (PNB) for lower extremity processes has resulted in breakthroughs in nerve localization, such as ultrasound imaging and continuous catheter technology. Consequently, we conducted a study in which we added infiltration between the popliteal artery and the capsule of the knee (iPACK) block to FNB and ACB to identify pain in the posterior part of the knee joint.

This study was designed to compare limb mobilization after 0.2% ropivacaine infusion in ACB with that after FNB used for postoperative pain relief in patients undergoing unilateral TKA. The secondary objectives of this study were to compare the effects of postoperative analgesics and assess the total rescue analgesic requirement within 24 hours after surgery in both groups.

## Materials and methods

Study site

The study was conducted in the Department of Anesthesiology, Max Super Speciality Hospital, Patparganj, New Delhi, after obtaining approval from the Institutional Ethical and Scientific Committee. The duration of this study was nine months, including the follow-up duration, after obtaining approval from the Scientific and Ethics Committee of the institution from October 2019 to June 2020.

Study population

We enrolled 50 American Society of Anesthesiologists (ASA) grades I-III individuals of either sex aged 35-75 years undergoing unilateral TKR surgery in our center after obtaining written consent from them. A computer-generated random sequence was used to randomize the patients into two groups, with each group comprising 25 individuals. Patients undergoing bilateral TKR but at two different sittings of one knee at a time at different calendar dates were treated as separate cases.

Study design

A prospective and randomized controlled study was conducted. The patients were split into two groups: ACB and FNB groups. The ACB group consisted of patients who received continuous ACB with iPACK block, whereas the FNB group consisted of patients who received continuous FNB with iPACK block.

Inclusion and exclusion criteria

ASA grade I-III patients aged between 35 and 75 years undergoing unilateral TKR were included in this study. Those who were allergic to local anesthetic agents, were unable to understand and use the numeric rating scale (NRS), had an infection at the needle puncture site, or had preexisting sensory or motor weakness were excluded from this study.

Study intervention

The steps of the intervention were as follows: (1) Patients were divided into two test groups: ACB and FNB. (2) Baseline vital signs were recorded. (3) General anesthesia was induced. (4) Ultrasound-guided iPACK block was applied before the start of surgery. (5) TKA was started, and vital signs were recorded every 15 minutes intraoperatively. (6) ACB, FNB, and subsequent catheter placement were performed in the immediate postoperative period after surgical completion. (7) In the iPACK block, a single 20-mL shot of 0.2% ropivacaine was administered, and no catheter was placed. (8) In the ACB, a single 20-mL shot of 0.2% ropivacaine was injected, and a catheter was placed. The continuous infusion solutions for ACB contained 0.2% plain ropivacaine, which was delivered at a rate of 3 mL/hour one hour after the surgery was started. (9) In the FNB, a single 20-mL shot of 0.2% ropivacaine was administered, and a catheter was placed. The continuous infusion solutions for FNB contained 0.2% plain ropivacaine, which was delivered at a rate of 3 mL/hour one hour after the surgery was started.

Statistical analysis

Sample size calculation was done on the basis of the Timed Up and Go (TUG) test in the FNB/ACB group in a study by Hegazy and Sultan [7]. These are reported as 10.3 ± 3.5 seconds and 5.2 ± 0.7 seconds on postoperative day one (POD1), respectively. To be able to detect a difference of at least one second on average, with a power of 80% and a significance level of 5%, the sample size was calculated to be 25 in each group according to the following formula:

n = (σ_1_^2 ^+ σ_^2^_^2^) . [Z_α/2_ + Z_β_]^2^/∂2

Where

σ_1 _ = 3.5; σ_2_^2^ = 0.7; Z _α/2 _ = 1.96 for 5% level of significance; Z _β _ = 0.84 for 80% power; ∂2 = 1 (the minimum difference to be detected).

Statistical Package for the Social Sciences version 17.0 (SPSS Inc., Chicago, IL, USA) was used to perform all statistical analyses. Continuous variables were compared between the groups using the Student’s t-test. Nominal categorical data between the groups were compared using the chi-square test or Fisher’s exact test, as appropriate. For all statistical tests, differences with p-values of less than 0.05 were considered statistically significant.

## Results

Demographic profile of the respondents

**Figure 1:**
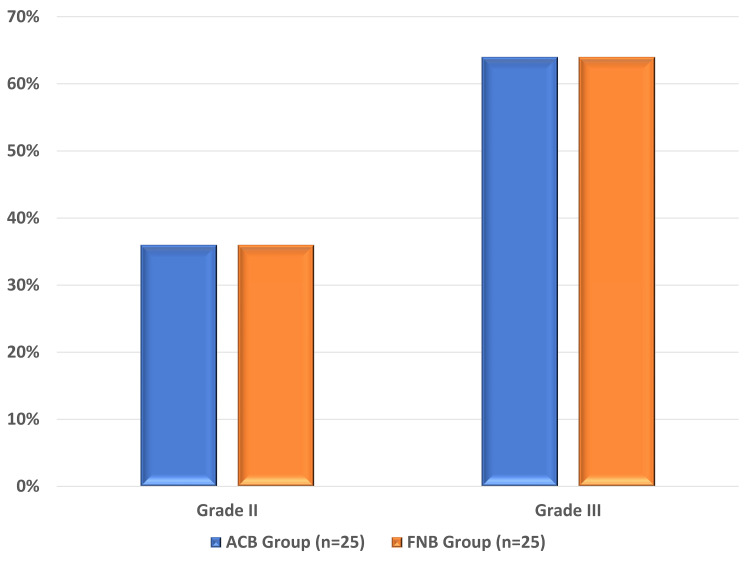
American Society of Anesthesiologists grading in both study groups. ACB: adductor canal block; FNB: femoral nerve block

Most patients in both groups belonged to the 61-70-year age group (56.0% in the ACB group and 40.0% in the FNB group), followed by the 51-60-year age group (32.0% in the ACB group and 40.0% in the FNB group). Of the 25 patients in the ACB Group, 11 (44.0%) were males and 14 (56.0%) were females; meanwhile, in the FNB group, eight (25.0%) were males and 17 (68.0%) were females, showing female dominance in both groups. No significant difference in sex distribution was observed between the two groups (p = 0.38). In both groups, of the 25 patients, 16 (64.0%) were classified as ASA grade III and the remaining nine (36.0%) were classified as ASA grade II. The mean age of the patients in the ACB group was 63.60 ± 6.08 years, whereas it was 63.52 ± 7.71 years in the FNB group. Regarding mean age distribution, gender, and ASA grading, both groups were comparable (Table 1 and Figure 1).

**Table 1:** Age and gender distribution of the study participants. ACB: adductor canal block; FNB: femoral nerve block; SD: standard deviation

	ACB group (n = 25)		FNB group (n = 25)	
	Number of patients	%	Number of patients	%
Age group				
51–60 years	8	32.0	10	40.0
61–70 years	14	56.0	10	40.0
>70 years	3	12.0	5	20.0
Mean age ± SD	63.60 ± 6.08		63.52 ± 7.71	
Gender				
Male	11	44	8	32
Female	14	56	17	68

Clinical diagnosis

At baseline, all hemodynamic variables, such as pulse rate, respiratory rate, SpO_2_, systolic blood pressure, and diastolic blood pressure, were not significantly different between the two groups (p > 0.05). At various intervals, we discovered that in the first 24 postoperative hours, no significant difference in the mean pulse rate (beats/minute) was observed between the ACB and FNB groups (p > 0.05). Moreover, in the first 24 postoperative hours, no significant difference in the mean systolic blood pressure was observed between the ACB and FNB groups (p > 0.05). No significant changes were observed in systolic blood pressure during the postoperative period in both groups, except at one hour after the operation. Furthermore, we found that the mean diastolic blood pressure did not change in the ACB and FNB groups for the entire postoperative period, except at one hour after the operation. No significant difference in the mean diastolic blood pressure was found in the first 24 hours following surgery between the two groups (p > 0.05) (Table 2).

**Table 2:** Systolic and diastolic blood pressure (mmHg) between both study groups at different intervals (Student’s t-test was used to compare blood pressure at different time intervals). ACB: adductor canal block; FNB: femoral nerve block; BP: blood pressure

Time interval	Systolic BP (mmHg)			Diastolic BP (mmHg)		
	ACB group (n = 25)	FNB group (n = 25)	P-value	ACB group (n = 25)	FNB group (n = 25)	P-value
Baseline	128.16 ± 12.22	124.08 ± 10.29	0.21	75.01 ± 8.03	73.68 ± 8.67	0.57
At 1 hour	136.36 ± 10.05	129.76 ± 9.53	0.02	84.40 ± 7.16	80.72 ± 5.56	0.04
At 2 hours	127.52 ± 7.79	125.04 ± 8.83	0.29	77.72 ± 5.84	77.48 ± 6.55	0.89
At 6 hours	124.76 ± 6.60	121.44 ± 7.54	0.10	75.20 ± 4.91	76.0 ± 7.90	0.67
At 12 hours	121.96 ± 6.25	119.04 ± 10.02	0.22	70.80 ± 4.97	73.64 ± 8.85	0.16
At 24 hours	120.20 ± 7.30	120.72 ± 8.67	0.82	69.76 ± 5.72	72.60 ± 9.32	0.20

Intervention during the postoperative period

When patients during the postoperative period were asked to do limb movements, all patients in the FNB group performed them 2-24 hours after surgery, whereas, in the ACB group, one patient could not move their limbs two hours after surgery; however, all patients could move their limbs from six hours onward after surgery (Table 3).

**Table 3:** Limb movement between both study groups at different intervals (the chi-square test was used to compare limb movement of the operated limb in both groups). ACB: adductor canal block; FNB: femoral nerve block

Movement of the operated limb	ACB group (n = 25)		FNB group (n = 25)		P-value
	Number of patients	%	Number of patients	%	
At 2 hours	24	96.0	25	100.0	0.99
At 6 hours	25	100.0	25	100.0	-
At 12 hours	25	100.0	25	100.0	-
At 24 hours	25	100.0	25	100.0	-

When the patients were asked to undertake TUG tests during the postoperative period, the patients in the ACB group performed all tests (i.e., get up time; 3-m walk time; return from 3 m; and 10-m walk time) considerably faster than those in the FNB group. The mean get-up time in the ACB group was 39.08 ± 5.53 seconds, whereas, in the FNB group, it was 44.92 ± 7.10 seconds (p < 0.01). The 3-m walk time was 123.16 ± 15.90 seconds in the ACB group and 134.68 ± 13.13 seconds in the FNB group (p < 0.01). The 10-m walk time was 221.24 ± 18.82 seconds in the ACB group and 245.24 ± 21.68 seconds in the FNB group (p < 0.001) (Table 4).

**Table 4:** TUG test time between both study groups (Student’s t-test was used to compare both groups). ACB: adductor canal block; FNB: femoral nerve block; TUG: Timed Up and Go

TUG test time	ACB group (n = 25)	FNB group (n = 25)	P-value
Get-up time (seconds)	39.08 ± 5.53	44.92 ± 7.10	<0.01
3-m walk time (seconds)	123.16 ± 15.90	134.68 ± 13.13	<0.01
Return from 3 m (seconds)	114.28 ± 13.69	129.44 ± 13.34	<0.001
10-m walk time (seconds)	221.24 ± 18.82	245.24 ± 21.68	<0.001

Postoperative pain in both groups was assessed using NRS at different time intervals. One hour after surgery, the NRS score in the ACB group was 2.80 ± 1.44, whereas that in the FNB group was 2.84 ± 1.44 (p = 0.88). Two hours after surgery, the NRS score in the ACB group was 3.0 ± 1.26, whereas that in the FNB group was 3.24 ± 1.36 (p = 0.46). Six hours after surgery, the NRS score in the ACB group was 2.84 ± 0.89, whereas that in the FNB group was 3.0 ± 1.35 (p =0.98). Twelve hours after surgery, the NRS score in the ACB group was 3.20 ± 1.53, whereas that in the FNB group was 2.68 ± 1.21 (p = 0.36). Twenty-four hours after surgery, the NRS score in the ACB group was 2.0 ± 0.64, whereas that in the FNB group was 1.84 ± 0.62 (p = 0.37). Throughout the postoperative period, no statistically significant differences in the NRS scores were observed between the two study groups (Table 5).

**Table 5:** Postoperative pain assessment between the two study groups at different intervals (the chi-square test was used to compare both groups). ACB: adductor canal block; FNB: femoral nerve block; NRS: numeric rating scale

NRS	ACB group (n = 25)	FNB group (n = 25)	P-value
At 1 hour	2.80 ± 1.44	2.84 ± 1.44	0.88
At 2 hours	3.0 ± 1.26	3.24 ± 1.36	0.46
At 6 hours	2.84 ± 0.89	3.0 ± 1.35	0.98
At 12 hours	3.20 ± 1.53	2.68 ± 1.21	0.36
At 24 hours	2.0 ± 0.64	1.84 ± 0.62	0.37

**Figure 2:**
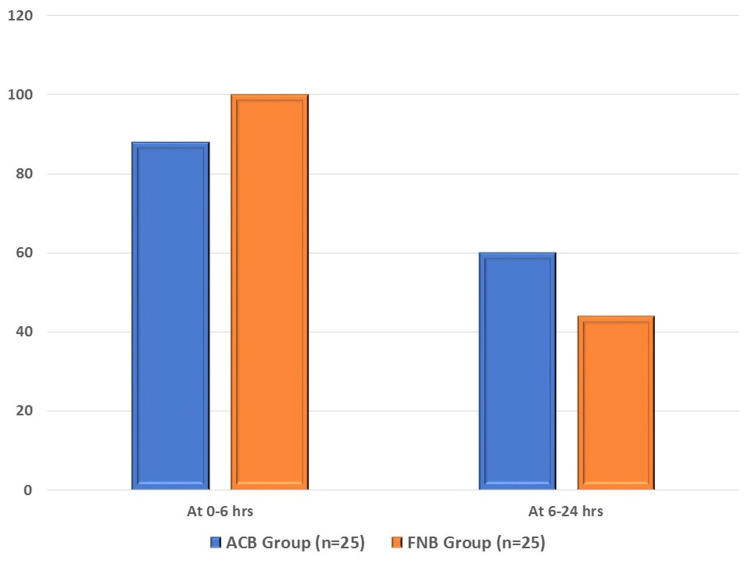
Requirement of rescue analgesia between the two study groups. ACB: adductor canal block; FNB: femoral nerve block

At 0-6 hours after surgery, the ACB group required 32.0 ± 21.42 µg of rescue analgesia, whereas the FNB group required 32.80 ± 9.79 µg. The ACB group required 15.60 ± 14.45 µg of analgesia 6-24 hours after surgery, whereas the FNB group required 10.80 ± 13.82 µg. Between the two groups, no statistically significant difference was observed (p > 0.05). In the ACB group, 22 (88.0%) of the 25 patients required emergency analgesia within six hours after surgery; however, in the FNB group, all 25 (100.0%) patients required rescue analgesia. Rescue analgesia was administered to 15 (60.0%) patients in the ACB group and 11 (44.0%) patients in the FNB group 6-24 hours after surgery (Figure 2).

**Figure 3:**
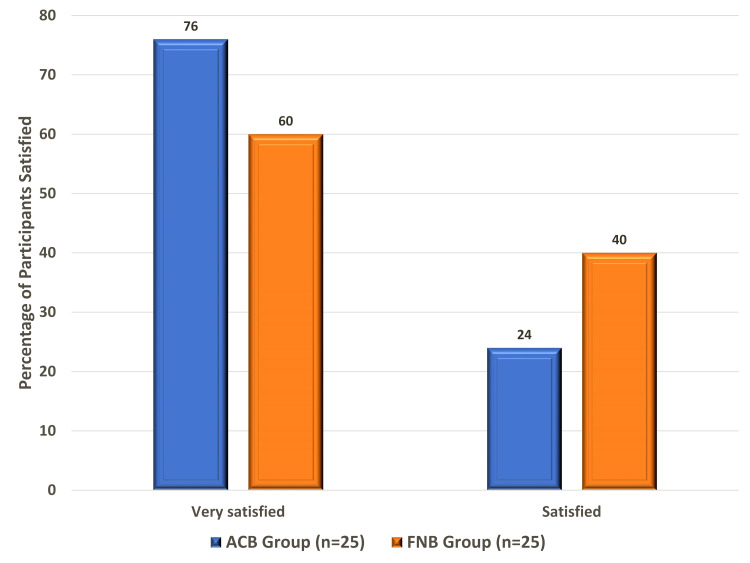
Patient satisfaction between the two study groups. ACB: adductor canal block; FNB: femoral nerve block

In the ACB group, more than three-quarters of the patients (76.0%) were extremely satisfied with the process and the remaining patients (24.0%) were satisfied, whereas, in the FNB group, 15 (60%) patients were extremely satisfied and the remaining 10 (40.0%) patients were satisfied. None of the patients in either group expressed dissatisfaction with the procedure (Figure 3).

## Discussion

We discovered that the intimate relation distributions of cases across the two groups were not markedly different (p = 0.38). A similar demographic pattern was reported by Elkassabany et al. [8] who also reported that the mean age was 63 ± 8 years in the ACB group and 65 ± 8 years in the FNB group with a female preponderance in both groups. Of the 31 patients in each group, 11 and 14 were classified as grade III in the ACB and FNB groups, respectively.

The value of rehabilitation has recently been increasing. A glass fiber outcome score for TKA called “discharge ready” has been proposed. It has the following four criteria: sufficient analgesia; intravenous drug problems; standing, walking 3 m, and sitting; and 30-m ambulation [9]. In addition to improving the surgical success of TKA, early ambulation helps reduce the occurrence of deep vein thrombosis in the legs [4] and improve muscular strength and walking control [5]. Patients who received ACB after TKA had superior ambulation length and TUG test results compared with those who received FNB, according to Grevstad et al. [10]. When Mudumbai et al. compared adductor canal continuous PNB with femoral continuous PNB, they found that it was linked to longer ambulation times on postoperative days one and two. To achieve early and effective rehabilitation following TKA, this rise is clinically significant. Clinically relevant quadriceps weakness has been found when extramedullary anesthetic infusion doses are administered through catheters implanted using routine methods according to the latest studies. Concerns have also been raised about a possible link between femoral spinal anesthesia and patient falls [6,11].

We discovered that the difference in the NRS scores between the two study groups was statistically quasi during the entire postoperative period, indicating that ACB is a good analgesic modality when matched with FNB after TKA. Because the femoral nerve commonly splits as it goes through the inguinal ligament, blocking the adductor canal must result in a more restricted distribution of sensory anesthesia and analgesia than FNB. Despite the fear that continuous ACBs could provide less analgesia, a direct assessment of pain levels revealed no difference between the two groups [12]. Jaeger et al. [13] examined how ACB and placebo affected pain in the first few days after TKA. While at rest and one hour after surgery, there was no correlation between pain visual analog scale (VAS) scores and physical flexion of the thigh or knee, when the area under the curve was determined again, they observed a significant decrease in pain VAS scores in favor of ACB during active knee flexion. Moreover, 25% of the patients might not get the study medication as scheduled 30 minutes after the last suture because of logistical problems, which could have left very little time for the blockade to have its best effect. ACB causes superior analgesia than FNB after TKA, according to Perlas et al. [14], although there was no change in analgesia induced using this approach compared with local infiltration analgesia (LIA) alone. Consequently, whether the analgesic advantages were due to ACB or LIA is unclear. Memtsoudis et al. have reported that 24 hours after surgery, more people who had the femoral method than the saphenous method had better anesthesia. In a simple comparison of methods, more patients showed better anesthesia using the femoral method than that using the saphenous method 24 hours after surgery, even though both kinds of blocks were related to similar outcomes. However, regarding pain intensity, the differences were statistically insignificant and most likely clinically unimportant.

To make the patient pain-free after this intensely painful process, pain management after TKA must be multimodal, powerful, and proactive. Patterson et al. [15] have identified common cumulative opioid consumption requirements in both groups, confirming our findings. Similarly, Mudumbai et al. could not find any significant difference in total opioid use between groups on postoperative days one or two. In this study, of the 25 patients, 22 (88.0%) needed rescue analgesia within six hours after surgery in the ACB group, whereas, in the FNB group, all 25 (100.0%) patients required rescue analgesia. Fifty-milligram tramadol in normal saline injections was administered to five patients in the FNB group and two patients in the ACB group in the study by Shah et al. [16].

An assessment of the general quality of pain management and the influence of treating pain on patients’ quality of rehabilitation and satisfaction should drive clinical therapy. In this study, we discovered that none of the subjects expressed dissatisfaction with the technique. Elkassabany et al. [8] found that the quality of recovery in both groups 24 hours, 48 hours, and one week after surgery was comparable. Developed for their use, the quality of recovery is a postoperative patient assessment system created by doctors. The quality of recovery and patient satisfaction were found to be linked in a study by Myles et al. [17] involving 10,811 individuals.

In both research populations, no such issues were found. In a comprehensive review, Koh et al. [18] concluded that ACB is a useful analgesic modality in modern perioperative care protocols, which focus on rapid recovery after knee surgery. For knee surgery, ACB is a straightforward procedure that can be performed with high success rates using recently available portable ultrasound technology. Moreover, compared with placebo, ACB offered great pain alleviation around the knee and helped maintain motor strength with few alterations from baseline. Many recent studies have shown that ACB has favorable analgesic effects and results in excellent mobility in patients who have undergone arthroscopic surgery or TKA. Considering these results and the existing perioperative protocol patterns toward rapid recovery following TKA, it appears that ACB should have been viewed in the context of a modern multimodal approach for managing pain following TKA.

## Conclusions

We conclude that the ACB/iPACK approach compared with the FNB/iPACK approach provides equal analgesia and improved early mobilization for unilateral TKR. For patients undergoing unilateral TKR in a standard hospital setting, ACB supplemented with an iPACK block is an effective alternative to continuous FNB. It provides comparable analgesia and early limb mobilizations at the end of 24 hours postoperatively.

We suggested that for patients undergoing unilateral TKR, ultrasound-guided ACB with iPACK block is a better choice for postoperative pain control compared to the FNB with iPACK. Moreover, we recommend conducting further studies on this method to judge its impact on the total duration of hospital stay, time to discharge, and overall hospital cost.
